# Leaf Photosynthesis and Its Temperature Response Are Different between Growth Stages and N Supplies in Rice Plants

**DOI:** 10.3390/ijms23073885

**Published:** 2022-03-31

**Authors:** Miao Ye, Zhengcan Zhang, Guanjun Huang, Yong Li

**Affiliations:** 1 Ministry of Agriculture and Rural Affairs Key Laboratory of Crop Ecophysiology and Farming System in the Middle Reaches of the Yangtze River, College of Plant Science and Technology, Huazhong Agricultural University, Wuhan 430070, China; mye@webmail.hzau.edu.cn (M.Y.); zhengcanzhang@outlook.com (Z.Z.); 2019301010004@webmail.hzau.edu.cn (G.H.); 2 Key Laboratory of Crop Genetics and Physiology of Jiangsu Province, Jiangsu Key Laboratory of Crop Cultivation and Physiology, Co-Innovation Center for Modern Production Technology of Grain Crops, Yangzhou University, Yangzhou 225009, China

**Keywords:** rice, photosynthesis, mesophyll conductance, stomatal conductance, temperature, leaf anatomy

## Abstract

Leaf photosynthesis is highly correlated with CO_2_-diffusion capacities, which are determined by both leaf anatomical traits and environmental stimuli. In the present study, leaf photosynthetic rate (*A*), stomatal conductance (*g*_s_), mesophyll conductance (*g*_m_) and the related leaf anatomical traits were studied on rice plants at two growth stages and with two different N supplies, and the response of photosynthesis to temperature (*T*) was also studied. We found that *g*_m_ was significantly higher at mid-tillering stage and at high N treatment. The larger *g*_m_ was related to a larger chloroplast surface area facing intercellular air spaces and a thinner cell wall in comparison with booting stage and zero N treatment. At mid-tillering stage and at high N treatment, *g*_m_ showed a stronger temperature response. The modelling of the *g*_m_-*T* relationships suggested that, in comparison with booting stage and zero N treatment, the stronger temperature response of *g*_m_ was related to the higher activation energy of the membrane at mid-tillering stage and at high N treatment. The findings in the present study can enhance our knowledge on the physiological and environmental determinants of photosynthesis.

## 1. Introduction

Rice is one of the most important cereal crops in the world, feeding more than half of the world’s population [1]. Studies relating to the physiological and anatomical determinants on rice photosynthesis, and to the response of photosynthesis to environmental stimuli, are of great importance to further improve rice yield. Photosynthesis in C3 plants, including rice plants, is limited by both CO_2_-diffusion capacities and biochemical functions [2,3,4,5,6,7]. Before being fixed by the key Calvin cycle enzyme of Rubisco, CO_2_ in the air should firstly diffuse across stomata to reach the substomatal cavity; thereafter, it will further diffuse across the cell wall, plasma membrane, cytoplasm and chloroplast envelope to reach the carboxylation sites [8,9]. The CO_2_-diffusion capacities through stomata and mesophyll cells are called stomatal conductance (*g*_s_) and mesophyll conductance (*g*_m_), respectively. Leaf photosynthesis has been frequently found to positively correlate with stomatal conductance and mesophyll conductance [10,11,12].

Both *g*_s_ and *g*_m_ are closely related to leaf anatomical traits. Previous studies have shown that stomatal conductance in rice plants is not correlated with either stomatal size or stomatal number [13,14], but it is significantly correlated with leaf hydraulic conductance (*K*_leaf_) [15]. The two major determinants of leaf hydraulic conductance are leaf vein density and xylem size [16,17,18], because more leaf veins can provide more parallel water flow paths through the vein system [19] and hydraulic conductance through leaf xylems is positively correlated with the xylem conduits diameter [18]. In rice plants, *K*_leaf_ is positively related to the area of xylem conduits within the bundle sheath, but it is not related to leaf vein density [15]. Both leaf vein density and xylem size can vary largely across different growth stages, which may in turn have a great effect on *g*_s_. Therefore, the first objective of this study was to investigate whether the variation in *g*_s_ across different growth stages is related to the variations in leaf vein density and xylem size.

Cell-wall thickness (*T*_cw_) and chloroplast surface area facing intercellular air spaces (*S*_c_) are two important leaf anatomical traits determining *g*_m_. It has been frequently found that *g*_m_ is negatively correlated with cell-wall thickness (*T*_cw_), and it is positively correlated with chloroplast surface area facing intercellular air spaces (*S*_c_) [7,20]. Leaf N content has a significant effect on *T*_cw_ and *S*_c_. In comparison with low N supply, high N supply can both decrease *T*_cw_ and increase *S*_c_, which are the major reasons for the increased *g*_m_ under high N supply [3,21]. There are large variations in *T*_cw_ and *S*_c_, which can subsequently have a large effect on *g*_m_. Therefore, the second objective of this study was to investigate the responses of *g*_m_, *T*_cw_ and *S*_c_ to growth stages under two different N supplies, which can improve our understanding on the determinants of *g*_m_.

Photosynthesis is sensitive to environmental variations, and temperature (*T*) is one of the major environmental stimuli that have great impacts on photosynthesis and crop production. However, it is not known whether the impact of temperature on photosynthesis varies across different growth stages, although it is known that N supply has a significant effect on the temperature response of photosynthesis in rice plants [22]. The variation in photosynthesis in response to temperature is largely related to mesophyll conductance [23], and the mechanisms relating to temperature response of *g*_m_ have been intensively studied [24,25,26,27]. CO_2_ diffusion through mesophyll cells can be divided into two processes, which are the liquid phase and membrane phase [25]. The liquid phase refers to CO_2_ diffusion through cell walls, cytoplasm and chloroplast stroma; the membrane phase refers to CO_2_ diffusion through plasma membrane and chloroplast envelope. It has been hypothesized that the sensitivity of mesophyll conductance to temperature (*E*_a,gm_) is determined by the activation energy of membrane (*E*_a,mem_) and by the ratio of CO_2_-diffusion conductance through liquid phase to that through membrane phase (*g*_liq_/*g*_mem_) [25]. The variations in leaf anatomical traits in response to growth stage and N supply may change the ratio of *g*_liq_/*g*_mem,_ and thus the sensitivity of mesophyll conductance to temperature. Therefore, the third objective of this study was to investigate the differential responses of *g*_m_ to temperature at different growth stages and N supplies.

In addition to *E*_a,mem_ and *g*_liq_/*g*_mem_, the response of leaf water potential (*Ψ*_leaf_) to temperature is also an important determinant in the response of *g*_m_ to temperature [27]. The increment of *g*_m_ with temperature can be substantially inhibited if *Ψ*_leaf_ decreases with temperature. It is not known whether *Ψ*_leaf_ can significantly decrease in response to increasing temperature at the late growth stage in rice plants, although *Ψ*_leaf_ is insensitive to temperature at tillering stage [23]. Therefore, the fourth objective was to investigate the variation in *Ψ*_leaf_ in response to the temperature, and its influence on the response of *g*_m_ to temperature.

To this end, a rice cultivar of Fengliangyouxiang 1 was grown in pots under two N supplies. At both tillering and booting stages, the gas-exchange parameters, the leaf anatomical traits and the temperature response of photosynthesis were investigated. We hypothesized that *T*_cw_ is significantly higher at booting stage than that at tillering stage, which in turn leads to a lower *g*_m_ and to a lower sensitivity of *g*_m_ to temperature at booting stage. The results will potentially improve our knowledge on rice photosynthesis, which will be beneficial to improving leaf photosynthesis and crop yields.

## 2. Results

### 2.1. Response of Leaf N Content to N Supplies and Growth Stages

**Figure 1:**
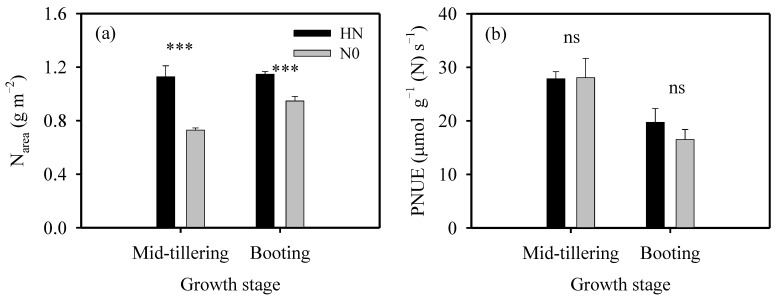
Area-based leaf N content (N_area_, (**a**)) and photosynthetic nitrogen use efficiency (PNUE, (**b**)) of plants under high N (HN) and zero N (N0) treatments at mid-tillering and booting stages. One-way analysis of variance (ANOVA) was used to assess the effect of N supply on parameters. ***, *p* < 0.001; ns, non-significant at *p* < 0.05 level.

Regardless of growth stages, area-based leaf N content (N_area_) under high N (HN) treatment was significantly higher than that under zero N (N0) treatment (Figure 1a). In comparison with N0 treatment, N_area_ under HN treatment was increased by 54.7% and 21.1% at mid-tillering and booting stages, respectively. Under HN treatment, N_area_ showed no obvious difference between the two growth stages; under N0 treatment, however, N_area_ at booting stage was 29.8% higher than that at mid-tillering stage.

### 2.2. The Responses of Gas-Exchange Parameters to Temperature under Different N Supplies and at Different Growth Stages

Net photosynthetic rate, stomatal conductance and mesophyll conductance at 25 °C were represented by *A*_25_, *g*_s,25_ and *g*_m,25_, respectively. At mid-tillering stage, *A*_25_, *g*_s,25_ and *g*_m,25_ under HN treatment were significantly higher than those under N0 treatment (Table 1). In comparison with N0 treatment, *A*_25_, *g*_s,25_ and *g*_m,25_ under HN treatment were increased by 55.8%, 74.6% and 101.5%, respectively. At booting stage, however, they were not significantly different between the two N treatments, although N_area_ under HN treatment was significantly higher than that under N0 treatment (Figure 1a). 

Under HN treatment, *A*_25_, *g*_s,25_ and *g*_m,25_ at mid-tillering stage were 67.4%, 113.9% and 135.7%, respectively, higher than those at booting stage (Table 1), although N_area_ was similar between the two growth stages under HN treatment (Figure 1a). Under N0 treatment, *A*_25_, *g*_s,25_ and *g*_m,25_ at mid-tillering stage were not significantly different with those at booting stage (Table 1), although N_area_ at mid-tillering stage was lower than that at booting stage (Figure 1a). Consequently, photosynthetic nitrogen-use efficiency (PNUE), which was calculated as the ratio of *A*_25_/N_area_, was significantly higher at mid-tillering stage than that at booting stage, although it was not significantly different between the two N treatments (Figure 1b).

**Figure 2:**
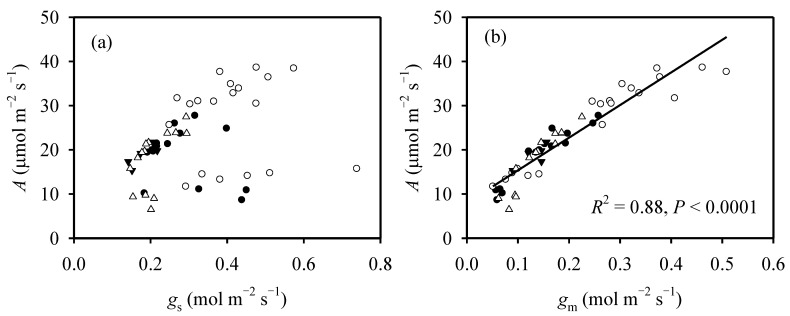
The relationships between net photosynthetic rate and (**a**) stomatal conductance and (**b**) mesophyll conductance across different temperatures under both N supplies and growth stages. *A*, net photosynthetic rate; *g*_s_, stomatal conductance; *g*_m_, mesophyll conductance. Filled cycles represent zero N (N0) treatment at mid-tillering stage, open cycles represent high N (HN) treatment at mid-tillering stage; filled triangles represent N0 treatment at booting stage, open triangles represent HN treatment at booting stage.

Both *A* and *g*_m_ increased dramatically with the increasing temperature; in contrast, temperature had no significant effect on *g*_s_ (Table 1). Across different temperatures, *A* was significantly correlated with *g*_m_, but it was not correlated with *g*_s_ (Figure 2). At mid-tillering stage, the modelled values of *E*_a,gm_ were 38.4 and 28.2 kJ mol^−1^ under HN and N0 treatments, respectively, and the modelled value at booting stage was 29.9 kJ mol^−1^ under HN treatment (Table 2). This suggested that *g*_m_ was less sensitive to temperature at N0 treatment and at booting stage.

Using the two-component model, the fitted membrane permeability to CO_2_ at 25 °C (*P*_mem,25_) varied from 0.722 mm s^−1^ to 1.272 mm s^−1^, and *E*_a,mem_ varied from 60.4 kJ mol^−1^ to 81.0 kJ mol^−1^ (Table 3), which fell well within the reported ranges [2,26]. The modelled *P*_mem,25_ and *E*_a,mem_ under HN treatment were larger than those under N0 treatment, and they were also larger at mid-tillering stage than those at booting stage.

### 2.3. Effects of N Supply and Growth Stage on Leaf Anatomical Traits

**Figure 3:**
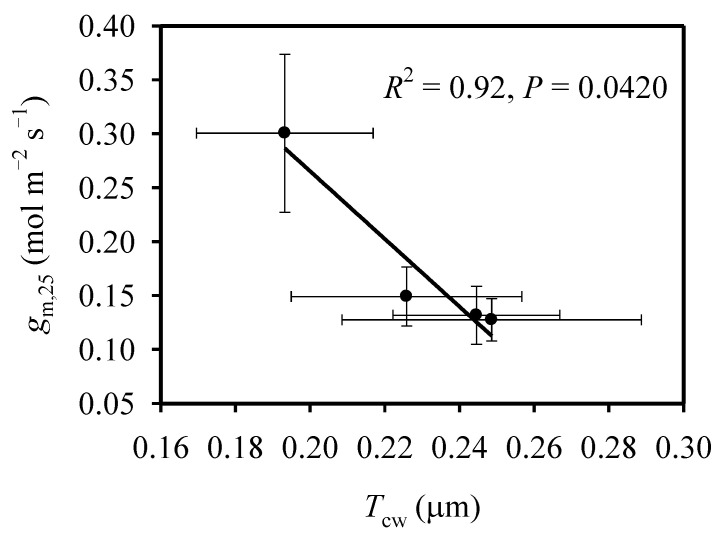
The relationship between mesophyll conductance at 25 °C (*g*_m,25_) and cell-wall thickness (*T*_cw_).

N supply had significant effects on *S*_c_ and *T*_cw_, but mesophyll cell surface area facing intercellular air spaces (*S*_m_) was not significantly different between N supplies (Table 4). In comparison with N0 treatment, *S*_c_ under HN treatment was increased by 25.0% and 21.8% at mid-tillering and booting stages, respectively. At mid-tillering stage, *T*_cw_ under HN treatment was significantly lower than that under N0 treatment; at booting stage, however, N supply had no significant effect on *T*_cw_. In comparison with mid-tillering stage, *S*_c_ was significantly lower, while *S*_m_ and *T*_cw_ were significantly higher at booting stage. Across different N supplies and growth stages, *g*_m,25_ was negatively correlated with *T*_cw_ (Figure 3), but it was not significantly related to either *S*_m_ or *S*_c_ (Figure S1).

### 2.4. The Responses of Leaf Hydraulic Traits to Temperature under Different N Supplies and at Different Growth Stages

Regardless of growth stage, temperature had no significant effect on *Ψ*_leaf_ under HN treatment; under N0 treatment, however, *Ψ*_leaf_ was more negative at 35 °C than that at 15 °C (Table 5). *Ψ*_leaf_ was not significantly different across different N supplies or growth stages. 

Regardless of growth stage, temperature had no significant effect on *K*_leaf_ (Table 5). In contrast, *K*_leaf_ under HN was significantly higher than that under N0 treatment. In comparison with N0 treatment, *K*_leaf_ under HN treatment was increased by 37.3% and 43.5% at mid-tillering and booting stages, respectively. Under both N supplies, *K*_leaf_ at mid-tillering stage was significantly higher than that at booting stage.

Leaf venation traits were significantly affected by N supplies and growth stages (Table S1). Regardless of growth stage, the inter-vein distance between major veins (IVD_major_) and the inter-vein distance between minor veins (IVD_minor_) under HN treatment were significantly higher than those under N0 treatment, although IVD_minor_ was not significantly different between different N supplies at mid-tillering stage. At mid-tillering stage, the area of xylem conduits in veins per leaf width (*S*_x_) under HN treatment was significantly lower than that under N0 treatment; at booting stage, however, *S*_x_ was not significantly different between two N supplies. In comparison with mid-tillering stage, IVD_major_, IVD_minor_ and *S*_x_ were all larger at booting stage. 

## 3. Discussion

The results obtained in the present study support our hypotheses that, in comparison with mid-tillering stage, *g*_m_ was lower and was less sensitive to temperature at booting stage. Moreover, *g*_m_ was significantly larger and was more sensitive to temperature under HN treatment than that under N0 treatment.

### 3.1. The Variation in Photosynthesis between Growth Stage and N Supply Is Related to CO_2_-Diffusion Capacity

The photosynthetic rate in C3 plants at an ambient CO_2_ concentration of ~400 μmol mol^−1^ is suggested to be limited by Rubisco carboxylation capacity, which is related to both the content and the specific activity of Rubisco [28,29], and the latter is dependent on CO_2_ partial pressure inside chloroplasts [3,21]. However, due to the significant resistance during the CO_2_-diffusion pathways through stomata and mesophyll cells, CO_2_ partial pressure inside chloroplasts is usually not saturated for C3 plants [24,30,31]. Therefore, CO_2_-diffusion capacities, namely *g*_s_ and *g*_m_, are major limitations to C3 photosynthesis [7,32]. In the present study, across different N treatments and growth stages, the variation trends of *g*_s_ and *g*_m_ were identical to that of net photosynthetic rate (Table 1). This suggested that the variation in photosynthesis between different growth stages and between different N supplies is also related to CO_2_-diffusion capacity.

The anatomical trait of *S*_c_ is an important determinant to *g*_m_ [33], and the value of *S*_c_ is related to chloroplast development and N supply [3,21]. The *S*_c_ value has been found to positively relate to N_area_ [34]. In the present study, however, the variations of *S*_c_ and N_area_ were uncoupled between two growth stages (Figure 1a and Table 4). The values of *S*_c_ at booting stage were significantly lower than those at mid-tillering stage (Table 4), although N_area_ at booting stage was similar to, or was even larger than, that at mid-tillering stage (Figure 1). The unparallel changes of *S*_c_ and N_area_ between different growth stages may be caused by differential N partitions. It is suggested that 10% of leaf N is distributed in cell walls [35], but the proportion can be increased to 30% in leaves with large leaf mass per area (LMA) and thick cell walls [36]. In the present study, *T*_cw_ was dramatically higher at booting stage than that at mid-tillering stage (Table 4). This suggested that N distribution to cell walls may be larger at booting stage than that at mid-tillering stage, which may in turn resulted in the unparallel changes of *S*_c_ and N_area_ between different growth stages.

In addition to *S*_c_, *T*_cw_ is also an important anatomical determinant in *g*_m_, which is negatively correlated with *T*_cw_ [2,20]. In line with previous studies [3,21], at mid-tillering stage, the larger *g*_m_ at HN treatment in comparison with N0 treatment can be explained by the increased *S*_c_ and the decreased *T*_cw_ (Table 1 and Table 4). In contrast, with a similar *T*_cw_ between two N supplies at booting stage, the larger *S*_c_ at HN treatment did not lead to an increased *g*_m_ in comparison to N0 treatment (Table 1 and Table 4). Moreover, *g*_m_ was significantly correlated with *T*_cw_ across different growth stages and N supplies (Figure 3), while it was not significantly correlated with *S*_c_ (Figure S1). This suggested that *T*_cw_ is a more important anatomical trait than *S*_c_ in determining *g*_m_, and the manipulation of cell walls is suggested to be an efficient approach to improve leaf photosynthesis [37]. In comparison with booting stage, the larger *g*_m_ at mid-tillering stage was related to the higher *S*_c_ and the lower *T*_cw_.

Stomatal conductance has been frequently found to be positively correlated with *K*_leaf_. In the present study, however, *g*_s_ was not correlated with either *K*_leaf_ or leaf venation traits (Table 1, Table 5 and Table S1). In the soil-plant-atmosphere continuum, the water diffusion resistance through leaves contributes ~30% of the whole plant hydraulic resistance [38]. In contrast, recent studies have suggested that root hydraulic resistance, including the resistance through radial pathway from root surface to the xylem and that through the root-soil interface, is the major resistance for water diffusion [39,40]. The relative resistance through leaves and roots in rice plants is not known, but we speculate that root hydraulic conductance may vary with growth stages and N supplies, which in turn determines the variation in *g*_s_.

### 3.2. Temperature Response of g_m_ Varies with Growth Stage and N Supply

Photosynthesis is sensitive to temperature, increasing with increasing temperature, but decreasing dramatically at supra-optimal temperature [27]. The increase in *A* with temperature is suggested to at least partially correlate with the increase in *g*_m_ [23]. In line with previous studies, in the present study, the response of *A* to temperature was similar to that of *g*_m_, while the response of *g*_s_ to temperature was different from both *A* and *g*_m_ (Table 1). This suggested that the response of photosynthesis to temperature is mainly driven by the *g*_m_-*T* relationship.

The *g*_m_-*T* relationship is correlated with the variation in *Ψ*_leaf_ in response to temperature [27], because leaf dehydration can severely depress *g*_m,_ probably through the deactivation of aquaporins [5,41]. Therefore, the decrease in *Ψ*_leaf_ in response to temperature can lead to a lower increment of *g*_m_ with temperature [27]. In the present study, however, there was little variation in *Ψ*_leaf_ in response to temperature, although *Ψ*_leaf_ was significantly decreased at N0 treatment at both growth stages (Table 5). This suggested that the differential sensitivity of *g*_m_ to temperature between growth stages and N treatments is not related to the variation in *Ψ*_leaf_.

The two-component model hypothesized that *g*_m_ would show a strong temperature response if *E*_a,mem_ were large and the ratio of *g*_liq,25_’/*g*_mem,25_’ were high [25], where *g*_liq,25_’ and *g*_mem,25_’ represent the CO_2_ conductance through the liquid phase per *S*_c_ and the CO_2_ conductance through the membrane phase per *S*_c_, respectively. The modelling of the *g*_m_-*T* relationships suggested that, in comparison with N0 treatment, *E*_a,mem_ was larger, while *g*_liq,25_’/*g*_mem,25_’ was lower at HN treatment; in comparison with booting stage, *E*_a,mem_ was larger while *g*_liq,25_’/*g*_mem,25_’ was comparable at mid-tillering stage (Table 3). This suggested that the larger *E*_a,mem_ was accounted for by the stronger temperature response of *g*_m_ at mid-tillering stage and at HN treatment. However, the factors that determine *E*_a,mem_ are not known, but the membrane compositions, such as cholesterol and aquaporins, are suggested to affect *E*_a,mem_ [26]. More research is needed to investigate the mechanisms underlying the differential *E*_a,mem_. 

## 4. Materials and Methods

### 4.1. Plant Materials and N Treatments

A rice cultivar of Fengliangyouxiang 1, which has been widely grown locally, was planted in pots in Huazhong Agricultural University (114.37° E, 30.48° N), Wuhan, Hubei province, China. After germination on moist filters on 15 July 2019, seeds were transferred to nursery boxes. When the seedlings had developed an average of three leaves, which usually requires 15 days, they were transplanted to 11.0 L pots with a density of three hills per pot and two seedlings per hill. Each pot was filled with 10.0 kg of soil. Phosphorus (P) and potassium (K) were applied as basal fertilizers at the rates of 1.50 and 1.89 g pot^−1^, respectively, in the form of KH_2_PO_4_. N was applied with urea at a rate of 1.60 g pot^−1^ at HN treatment, 40% of which was applied as the basal fertilizer, and another two topdressings of 30% each were applied at mid-tillering and booting stages. No N was applied for N0 treatment. The soil used in this study had the following properties: pH 7.1, 6.7 g kg^−1^ of organic matter, 6.27 mg kg^−1^ of Olsen-P, 129 mg kg^−1^ of exchangeable K, and 0.63‰ total N. There were 10 pots per treatment. Plants were irrigated daily with tap water, and a minimum 2 cm water layer was maintained to avoid drought stress. Measurements were conducted on the newest fully expanded leaves at mid-tillering stage and on the flag leaves at booting stage. 

### 4.2. Gas-Exchange Measurements

To minimise the effects of environmental fluctuations and midday depression on photosynthesis, rice plants were transferred to an environmentally controlled growth chamber (Conviron GR48, Controlled Environments Ltd., Winnipeg, MB, Canada) in the afternoon before the day of measurement. The air temperatures of the growth chamber were controlled to match the desired leaf temperatures of 15, 25 or 35 °C. The CO_2_ concentration and light intensity in the growth chamber were controlled at 400 μmol mol^−1^ and 1000 μmol m^−2^ s^−1^, respectively. A portable photosynthesis system (Licor-6800; Li-Cor Inc., Lincoln, NE, USA) with an integrated fluorescence leaf chamber (6800-01A) was used to measure leaf gas exchange and chlorophyll fluorescence between 08:00 and 16:00. Before the measurement, the leaves were attached to the leaf chamber to stabilize. CO_2_ concentration inside the leaf chamber was controlled to 400 µmol mol^−1^, and photosynthetic photon flux density (PPFD) was set to 1500 µmol m^−2^ s^−1^. The vapour-pressure deficit between leaf and air (VPD) increased dramatically with temperature. When leaf photosynthetic parameters were stabilised, which usually takes 15–25 min, gas-exchange parameters and chlorophyll fluorescence were simultaneously recorded with a light saturating pulse of 8000 µmol m^−2^ s^−1^. The actual photochemical efficiency of photosystem II (Φ_PSII_) was calculated as follows:

(1) $$\Phi_{\mathrm{PSII}}=\frac{F_{m}\prime -F_{s}}{F_{m}\prime }$$

where *F*_s_ and *F*_m_′ are steady-state fluorescence and the maximum fluorescence, respectively. The electron transport rate (*J*) was calculated as follows:

(2) $$J=PPFD\times \alpha \times \beta \times \Phi_{\mathrm{PSII}}$$

where α is the leaf absorptance and β is the partitioning of absorbed quanta between photosystem II and photosystem I. The product α × β was determined from the slope of the relationship between Φ_PSII_ and the quantum efficiency of CO_2_ uptake (Φ_CO_2__), which was measured by varying light intensity under non-photorespiratory conditions at <2% O_2_ [42]. 

The variable *J* method described in Harley et al. [43] was used to calculate chloroplastic CO_2_ concentration (*C*_c_) and *g*_m_:

(3) $$C_{c}=\frac{\Gamma *\times (J+8*(A+R_{d}))}{J-4*(A+R_{d})}$$

(4) $$g_{m}=\frac{A}{C_{i}-C_{c}}$$

where *C*_i_ is the intercellular CO_2_ concentration, Γ* is the CO_2_ compensation point in the absence of day respiration, and *R*_d_ is the day respiration rate. When calculating *g*_m_ at different temperatures, the values of Γ* and *R*_d_ at different temperatures were calculated using the following equation:

(5) $$P=e^{\left(c-\frac{E_{a}}{R\times (273+T)}\right)}$$

where *P* is calculated parameter, c is the scaling factor, *E*_a_ is the activation energy and *R* is the molar gas constant of 8.314 J K^−1^ mol^−1^. The values of c and *E*_a_ for Γ* and *R*_d_ were taken from Bernacchi et al. [24,44]. The values of c and *E*_a_ for Γ* are 13.49 and 24.46 kJ mol^−1^, respectively, while for *R*_d,_ they are 18.72 kJ mol^−1^ and 46.39 kJ mol^−1^, respectively. At booting stage, gas-exchange measurements under N0 treatment were conducted at 25 °C only (Table 1). The leaf photosynthetic rate, stomatal conductance and mesophyll conductance at 25 °C are represented by *A*_25_, *g*_s,25_ and *g*_m,25_, respectively.

### 4.3. Measurements of Leaf Hydraulic Parameters

After gas-exchange measurements were complete, the leaves were immediately detached and placed in a previously exhaled-in and sealable bag. After equilibration for at least 10 min, *Ψ*_leaf_ was measured using a pressure chamber (PMS Instrument Company, Albany, OR, USA).

The measurements of *K*_leaf_ at different temperatures were conducted in the same growth chamber that was used for the gas-exchange measurements. The environments during the measurement of *K*_leaf_ were similar to those used for the gas-exchange measurements. *K*_leaf_ was measured using the evaporating flux method (EFM) [45]. Briefly, the newest fully expanded leaves were excised with a fresh razor blade, and then immediately recut under water. Then, the leaf was connected to silicone tubing with a compression fitting under water to prevent air entering the system. The tubing connected the leaf to a hard tube connected to a graduated cylinder on a balance capable of reading 0.1 mg. The balance logged data every 30 s to a computer. The excised leaves were placed under LED lights for transpiration; the PPFD at the leaf level was 1500 μmol m^−2^ s^−1^. After equilibration to a steady state, which required ∼30 min after excising the leaves, leaf transpiration rate (*T*_r_) was calculated after measuring the leaf area. Afterwards, the leaves were immediately placed in a previously exhaled-in and sealable bag. After equilibration for at least 20 min, *Ψ*_leaf_ was measured using a pressure chamber. The unnormalised *K*_leaf_ (*K*_leaf_′) was calculated as follows [46]:

(6) $$K_{\mathrm{leaf}}\prime =\frac{T_{r}}{0-\Psi_{\mathrm{leaf}}}$$

During the measurement, leaf temperature was measured using a Multi-channel Digital Thermometer (AZ88598, AZ Instrument Corp. Ltd., Taichung, China). Water viscosity has significant effects on leaf hydraulic traits [47,48]. To exclude these effects, leaf hydraulic conductance at different temperatures were normalised to the water viscosity at 25 °C:

(7) $$K_{\mathrm{leaf}}=K_{\mathrm{leaf}}\prime \times \frac{\mu }{\mu_{25}}$$

where *μ* is the water viscosity at the measured leaf temperature and *μ*_25_ is the water viscosity at 25 °C.

### 4.4. Leaf N Content Measurement

Immediately after the gas-exchange measurements, newly expanded leaves were detached to measure leaf area using a LI-Cor 3000C (LI-COR Inc., Lincoln, NE, USA) leaf area analyser. Then, the leaves were oven-dried at 80 °C to reach a constant weight. Afterwards, leaf dry mass was weighed, and LMA was calculated as the ratio of leaf dry mass to leaf area. Mass-based leaf N content (N_mass_, %) was measured using a stable isotope ratio mass spectrometer (IsoPrime 100, IRMS, Isoprime Ldt., Cheadle, UK), and N_area_ was calculated as: N_area_ = N_mass_ × LMA. PNUE was calculated as: PNUE = *A*_25_/N_area_.

### 4.5. Measurements of Leaf Anatomical Traits

Paraffin and ultrathin sections were made from three leaves per treatment to analyse leaf anatomy using light microscope (LM) and transmission electron microscope (TEM). For the paraffin sections, leaf discs of about 5.0 mm length were cut from the middle of the leaves, and they were then fixed in FAA buffer (5% formaldehyde, 5% glacial acetic acid and 63% alcohol (*v*/*v*) in pure water) at 4 °C for 24 h. Thereafter, they were vacuumed in a vacuum chamber (DZF-6050, Shanghai Hasuc Co., Ltd., Shanghai, China). The samples were embedded in paraffins, and the leaf cross-sections were made by professionals from Wuhan Google Biotechnology Co. Ltd. The paraffin sections were stained with safranin-fast green, and they were photographed at a magnification of ×300 with a Nikon Eclipse E100 LM (Nikon Optical, Tokyo, Japan). There were 6–9 LM images taken for each treatment. The *S*_x_, IVD_major_ and IVD_minor_ were measured using the ImageJ software.

For the ultrathin sections, small leaf sections of 2.0 × 2.0 mm were cut from middle of the leaves (avoiding midrib). The leaf sections were infiltrated with fixative 2.5% (*v*/*v*) glutaric aldehyde in 0.1 M phosphate buffer (pH = 7.6) in a vacuum chamber for 2 h. Ultrathin sections were made from Wuhan Google Biotechnology Co. Ltd. Images were acquired using a transmission electron microscope (H-7650; Hitachi-Science & Technology, Tokyo, Japan). The LM and TEM images (Figures S2 and S3) were used to measure *S*_c_ and *S*_m_ following the methods described in Evans et al. [34] and Ye et al. [49]. Cell-wall thickness was measured with ×10,000 TEM images using the ImageJ software.

### 4.6. Quantification of the Temperature Response of g_m_

The response of *g*_m_ to temperature was modelled using the equation:

(8) $$g_{m}=\frac{e^{\left(c-\frac{E_{a,\mathrm{gm}}}{R\times T_{k}}\right)}}{1+e^{\left(\frac{\Delta S\times T_{k}-E_{d,gm}}{R\times T_{k}}\right)}}$$

where ΔS is an entropy term and *E*_d,gm_ is a term for deactivation of *g*_m_ [24].

### 4.7. Modelling the Temperature Response of g_m_

In order to interpret the difference in the *g*_m_-*T* relationships between growth stages and N supplies, we used the equations in von Caemmerer and Evans [26] to model the temperature response of *g*_m_. Generally, *g*_m_ was separated into liquid phase and membrane phase:

(9) $$g_{m}=\frac{1}{\frac{1}{g_{\mathrm{liq}}}+\frac{1}{g_{\mathrm{mem}}}}$$

The CO_2_ conductance through the liquid phase per *S*_c_ (gliq′=gliqSc) can be given by

(10) $$g_{\mathrm{liq}}\prime =\frac{\rho HD}{l}$$

where *ρ* (mol m^−3^) is the molar density of water, H (bar^−1^) is the Henry coefficient for CO_2_, D (m^2^ s^−1^) is the diffusivity of CO_2_ in water and *l* (m) is the effective pathlength.

Solubility of CO_2_ in water decreases with temperature and

(11) $$\rho H=33.06\times e^{\left(2400\times (\frac{1}{273+T}-\frac{1}{298})\right)}$$

Diffusivity of CO_2_ in water increases with temperature:

(12) $$D=1.81\times 10^{-6}\times e^{\left(-\frac{16900}{R(273+T)}\right)}$$

*l* was calculated from *T*_cw_ and cell-wall porosity (*P*_cw_) [50]:

(13) $$l=\frac{T_{cw}}{P_{cw}}$$

*P*_cw_ varied with *T*_cw_ according to Tosens et al. [51]:

(14) $$P_{cw}=-0.3733\times T_{cw}+0.3378$$

The temperature dependence of CO_2_-diffusion across biological membranes per *S*_c_ (gmem′=gmemSc) is assumed to be exponential:

(15) $$g_{\mathrm{mem}}\prime =\rho HP_{mem,25}\times e^{\frac{(T-25)\times E_{a,men}}{R\times 298\times (273+T)}}$$

### 4.8. Statistical Analysis

One-way and two-way analyses of variance (ANOVA) were used to assess the effects of leaf temperature, N supply and growth stage, as well as their interactions on parameters using Statistix 9.0 software (Analytical Software, Tallahassee, FL, USA). Parameters were compared between treatments based on the least significant difference (LSD) test level at the 0.05 probability level. Graphs were created, and a linear regression analysis was performed to test the correlations between parameters using SigmaPlot 10.0 (Systat Software Inc., San Jose, CA, USA).

## 5. Conclusions

Leaf photosynthesis can be significantly affected by growth stage and N supply, and the larger photosynthetic rate at mid-tillering stage and with HN treatment was related to the higher *g*_s_ and *g*_m_. In comparison with *S*_c_, *T*_cw_ is a more important anatomical trait in determining *g*_m_ at different growth stages. The response of leaf photosynthesis to temperature can also be affected by growth stage and N supply, and these effects are related to the strong temperature response of *g*_m_. The stronger response of *g*_m_ to temperature at mid-tillering stage and HN treatment was related to the larger *E*_a,mem_.

## Figures and Tables

**Table 1:** Temperature responses of leaf gas-exchange parameters under different nitrogen (N) supplies at two growth stages.

Growth Stage	N Supply	Leaf Temperature (°C)	*A* (µmol m^−2^ s^−1^)	*g*_s_ (mol m^−2^ s^−1^)	*g*_m_ (mol m^−2^ s^−1^)
Mid-tillering	N0	15	10.2 ± 1.1 c	0.350 ± 0.124 a	0.063 ± 0.006 c
		25	20.3 ± 1.0 b	0.213 ± 0.020 b	0.149 ± 0.027 b
		35	24.6 ± 2.6 a	0.294 ± 0.068 ab	0.207 ± 0.043 a
	HN	15	14.0 ± 1.4 b	0.452 ± 0.161 a	0.096 ± 0.032 c
		25	31.7 ± 4.1 a	0.372 ± 0.087 a	0.300 ± 0.073 b
		35	35.1 ± 2.7 a	0.430 ± 0.105 a	0.388 ± 0.066 a
Booting	N0	15	-	-	-
		25	18.7 ± 2.5	0.178 ± 0.174	0.132 ± 0.121
		35	-	-	-
	HN	15	8.6 ± 1.5 c	0.188 ± 0.024 b	0.084 ± 0.016 c
		25	18.9 ± 2.2 b	0.174 ± 0.018 b	0.127 ± 0.019 b
		35	24.0 ± 2.2 a	0.257 ± 0.044 a	0.188 ± 0.021 a
ANOVA					
T			***	ns	***
N			**	ns	*
S			***	**	*
T × N			**	ns	***
T × S			**	ns	***
N × S			***	*	***

Data are means ± SD of 4–7 replicates. *, *p* < 0.05; **, *p* < 0.01; ***, *p* < 0.001; ns, non-significant. T, temperature; S, growth stage; *A*, net photosynthetic rate; *g*_s_, stomatal conductance; *g*_m_, mesophyll conductance. The data followed by different letters in the same N and growth stages are significant at *p* < 0.05 level.

**Table 2:** The modelled parameters using Equation (8) for the temperature response of mesophyll conductance (*g*_m_).

Growth Stage	N Supply	*c*	*E*_a,gm_ (kJ mol^−1^)	ΔS (kJ K^−1^ mol^−1^)	*E*_d,gm_ (kJ mol^−1^)
Mid-tillering	N0	11.2	28.2	1.25	437.4
	HN	15.3	38.4	1.25	437.4
Booting	N0	-	-	-	-
	HN	12.1	29.9	1.25	437.4

*c*, a scaling factor; *E*_a,gm_, activation energy of *g*_m_; ΔS, an entropy term; *E*_d,gm_, deactivation energy of *g*_m_.

**Table 3:** The modelled parameters using the two-component model for the temperature response of mesophyll conductance (*g*_m_) under two N supplies at different growth stages.

Growth Stage	N Supply	*l* (µm)	*P*_mem,25_ (mm s^−1^)	*E*_a,mem_ (kJ mol^−1^)	*g*_liq,25_’ (mol CO_2_ m^−2^ s^−1^)	*g*_mem,25_’ (mol CO_2_ m^−2^ s^−1^)	*g*_m,25_’ (mol CO_2_ m^−2^ s^−1^)	*g*_liq,25_’/*g*_mem,25_’
Mid-tillering	N0	0.89	0.722	60.4	0.0732	0.0239	0.0180	3.07
	HN	0.73	1.272	81.0	0.0897	0.0421	0.0286	2.13
Booting	N0	-	-	-	-	-	-	-
	HN	1.01	0.910	66.4	0.0643	0.0301	0.0205	2.14

*l*, effective pathlength; *P*_mem,25_, combined membrane permeability to CO_2_ at 25 °C; *E*_a,mem_, activation energy of CO_2_-diffusion conductance through the membrane; *g*_liq,25_’, CO_2_-diffusion conductance through the liquid phase per surface areas of chloroplasts facing the intercellular air spaces at 25 °C; *g*_mem,25_’, CO_2_-diffusion conductance through the membrane per surface areas of chloroplasts facing the intercellular air spaces at 25 °C; *g*_m,25_’, mesophyll conductance at 25 °C per surface areas of chloroplasts facing the intercellular air spaces.

**Table 4:** Leaf anatomical traits under different N supplies at two growth stages.

Growth Stage	N Supply	*S*_m_ (µm^2^ µm^−2^)	*S*_c_ (µm^2^ µm^−2^)	*T*_cw_ (µm)
Mid-tillering	N0	10.95 ± 1.70 a	6.92 ± 1.82 b	0.226 ± 0.030 a
	HN	10.95 ± 1.51 a	8.64 ± 1.90 a	0.193 ± 0.024 b
Booting	N0	11.37 ± 1.01 a	5.25 ± 0.97 b	0.245 ± 0.022 a
	HN	11.69 ± 0.98 a	6.40 ± 1.76 a	0.249 ± 0.042 a
ANOVA				
N		ns	**	*
S		**	***	***
N × S		ns	ns	*

*, *p* < 0.05; **, *p* < 0.01; ***, *p* < 0.001; ns, non-significant. S, growth stage; *S*_m_, the mesophyll cell surface area facing intercellular airspace per leaf area; *S*_c_, the surface area of chloroplasts facing intercellular airspace per leaf area; *T*_cw_, cell-wall thickness. The data followed by different letters in the same growth stages are significant at *p* < 0.05 level.

**Table 5:** Temperature responses of leaf hydraulic parameters under two N supplies at different growth stages.

Growth Stage	N Supply	Leaf Temperature (°C)	*Ψ*_leaf_ (MPa)	*K*_leaf_ (mmol m^−2^ s^−1^ MPa^−1^)
Mid-tillering	N0	15	−0.293 ± 0.087 a	8.57 ± 1.11 a
		25	−0.285 ± 0.090 a	7.86 ± 1.80 a
		35	−0.423 ± 0.097 b	7.05 ± 1.56 a
	HN	15	−0.360 ± 0.099 a	11.39 ± 2.53 a
		25	−0.430 ± 0.114 a	10.71 ± 1.45 a
		35	−0.370 ± 0.106 a	10.13 ± 2.58 a
Booting	N0	15	−0.155 ± 0.064 a	7.48 ± 2.25 a
		25	−0.278 ± 0.068 b	5.98 ± 1.78 a
		35	−0.560 ± 0.140 c	6.03 ± 1.55 a
	HN	15	−0.395 ± 0.137 a	8.83 ± 1.16 a
		25	−0.310 ± 0.081 a	9.20 ± 2.17 a
		35	−0.425 ± 0.044 a	9.59 ± 1.38 a
ANOVA				
T			***	ns
N			ns	***
S			ns	**
T × N			***	ns
T × S			*	ns
N × S			ns	ns

Data are means ± SD of 4–7 replicates. *, *p* < 0.05; **, *p* < 0.01; ***, *p* < 0.001; ns, non-significant. S represents growth stage. *Ψ*_leaf_, leaf water potential; *K*_leaf_, the normalised leaf hydraulic conductance with water viscosity. The data followed by different letters in the same N and growth stages are significant at *p* < 0.05 level.

## Supplementary Materials

The following supporting information can be downloaded at: https://www.mdpi.com/article/10.3390/ijms23073885/s1.
