# Supplementary material for: Leaf Photosynthesis and Its Temperature Response Are Different between Growth Stages and N Supplies in Rice Plants
Source: Int J Mol Sci. 2022 Mar 31;23(7):3885. doi: 10.3390/ijms23073885 (PMC8999464; doi:10.3390/ijms23073885)
Supplement: Supplementary file 1 [file ijms-23-03885-s001.zip › ijms-1635187-Supplementary.pdf]

Table S1. Effects of N supplies and growth stages on leaf morphological and xylem anatomical traits.

Growth stage	N treatment	S_x ($\mu m^2\ \mu m^{-1}$)	IVD_{major} (mm)	IVD_{minor} (mm)
Mid-tillering	N0	3.62 ± 0.21 a	1.07 ± 0.02 b	0.17 ± 0.01 a
	HN	3.29 ± 0.16 b	1.40 ± 0.15 a	0.17 ± 0.01 a
Booting	N0	3.90 ± 0.42 a	1.15 ± 0.11 b	0.18 ± 0.01 b
	HN	3.78 ± 0.18 a	1.48 ± 0.11 a	0.23 ± 0.01 a
ANOVA				
N		**	***	***
S		***	*	***
N×S		ns	ns	***

*, $p < 0.05$; **, $p < 0.01$; ***, $p < 0.001$; ns, non-significant. S represents growth stage. S_x, area of xylem conduits in veins per leaf width; IVD_{major}, inter-vein distance between major veins; IVD_{minor}, inter-vein distance between minor veins. The data followed by different letters in the same growth stages are significant at $p < 0.05$ level.

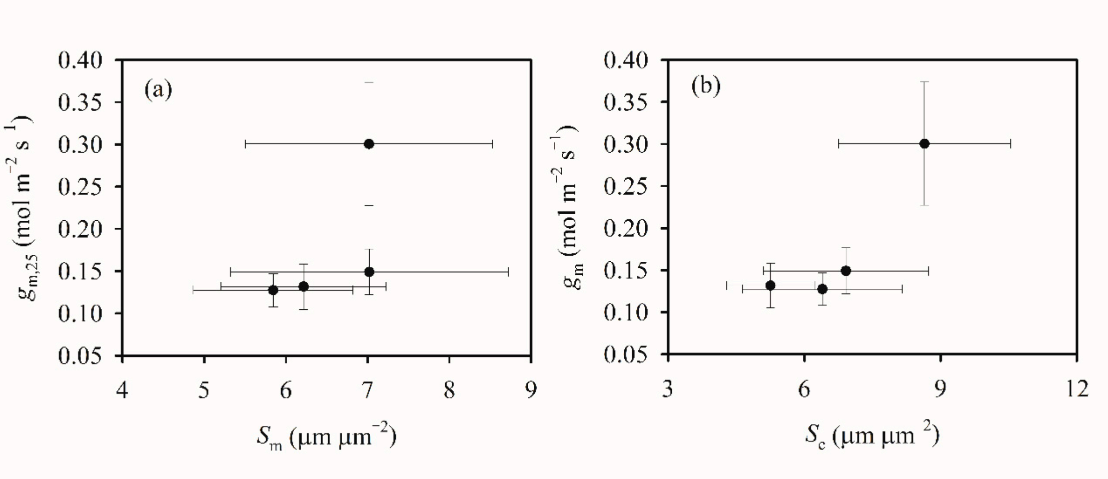

Figure S1. The relationships between mesophyll conductance at 25 °C ($g_{m,25}$) and (a) the surface area of mesophyll cell facing intercellular airspace per leaf area (S_m) and (b) the surface area of chloroplasts facing intercellular airspace per leaf area (S_c).

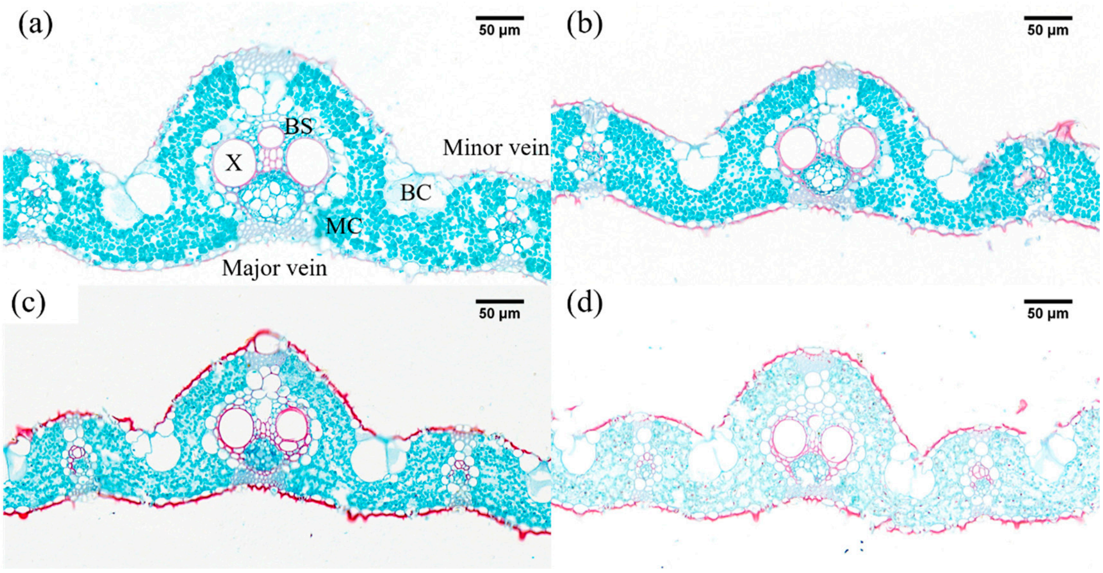

Figure S2. Light microscope (LM) images of rice leaves under two N supplies at mid-tillering (a, b) and booting stages (c, d). The panels of a and c are LM images under high N supply, and the panels of b and d are LM images under zero N treatment. X, xylem; BS, bundle sheath; BC, bulliform cell; MC, mesophyll cell.

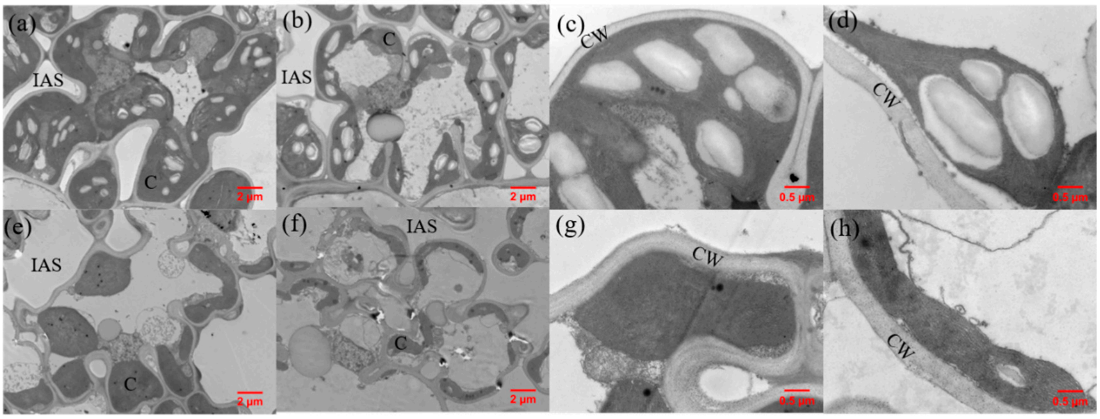

Figure S3. Transmission electron microscope (TEM) images of rice leaves under two N supplies at mid-tillering (a-d) and booting (e-h) stages. The panels of a, b, e and f are ×2500 TEM images, and the panels of c, d, g and h are ×10000 TEM images. The panels of a, c, e and f are TEM images under high N supply, and the panels of b, d, f and h are TEM images under zero N supply. IAS, intercellular air space; CW, cell wall; C, chloroplast.
